# Machine learning is a valid method for predicting prehospital delay after acute ischemic stroke

**DOI:** 10.1002/brb3.1794

**Published:** 2020-08-18

**Authors:** Li Yang, Qinqin Liu, Qiuli Zhao, Xuemei Zhu, Ling Wang

**Affiliations:** ^1^ School of Nursing Qingdao University Qingdao China; ^2^ School of Nursing The second Affiliated Hospital of Harbin Medical University Harbin Medical University Harbin China

**Keywords:** acute ischemic stroke, Bayesian network, machine learning, prehospital delay, support vector machine

## Abstract

**Objectives:**

This study aimed to identify the influencing factors associated with long onset‐to‐door time and establish predictive models that could help to assess the probability of prehospital delay in populations with a high risk for stroke.

**Materials and Methods:**

Patients who were diagnosed with acute ischemic stroke (AIS) and hospitalized between 1 November 2018 and 31 July 2019 were interviewed, and their medical records were extracted for data analysis. Two machine learning algorithms (support vector machine and Bayesian network) were applied in this study, and their predictive performance was compared with that of the classical logistic regression models after using several variable selection methods. Timely admission (onset‐to‐door time < 3 hr) and prehospital delay (onset‐to‐door time ≥ 3 hr) were the outcome variables. We computed the area under curve (AUC) and the difference in the mean AUC values between the models.

**Results:**

A total of 450 patients with AIS were enrolled; 57 (12.7%) with timely admission and 393 (87.3%) patients with prehospital delay. All models, both those constructed by logistic regression and those by machine learning, performed well in predicting prehospital delay (range mean AUC: 0.800–0.846). The difference in the mean AUC values between the best performing machine learning model and the best performing logistic regression model was negligible (0.014; 95% CI: 0.013–0.015).

**Conclusions:**

Machine learning algorithms were not inferior to logistic regression models for prediction of prehospital delay after stroke. All models provided good discrimination, thereby creating valuable diagnostic programs for prehospital delay prediction.

## Graphical abstract

This study found that the patient's knowledge about time window of intravenous thrombolysis for stroke was the strongest predictor of prehospital delay. Logistic regression, Bayesian network, and support vector machine models for predicting prehospital delay provided good discrimination, thus creating a valuable diagnostic procedure for prehospital delay prediction in high‐risk groups for stroke.

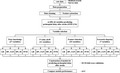

## 1 INTRODUCTION

The Global Burden of Diseases (GBD) 2017 study listed stroke as one of the leading causes of death and adult disability worldwide; the percent of deaths and disability‐adjusted life years of stroke in 2017 were 11.02% (ranked second) and 5.29% (ranked third), respectively (GBD, 2017 Causes of Death Collaborators, 2018). The China's Ministry of Health survey showed that ischemic stroke accounted for 77.8% of strokes (Wang et al., 2017). It has been proven that intravenous thrombolysis with recombinant tissue plasminogen activator (rt‐PA) is a useful method for preventing death, reducing irreversible brain damage, and improving the long‐term prognosis in patients with acute ischemic stroke (AIS; Wardlaw, Murray, Berge, & del Zoppo, 2014). However, it is only effective when given within a limited time after stroke onset (Emberson et al., 2014; Lees et al., 2016). Schwamm et al. (2013) reported that only 7.0% of patients with AIS in America were treated with rt‐PA. In addition, a research in Australia reported that only 14.7% of the patients who arrived early received thrombolytic therapy (Ashraf, Ines, Christopher, Rabsima, & Beata, 2013). According to the Chinese National Stroke Registry, only 1.6% of the patients with acute strokes received rt‐PA (Wang et al., 2011).

Previous systematic reviews reported that prehospital delay (from the onset of symptoms or the last known time without symptoms to the arrival at the hospital) accounted for the majority of treatment delay (Miriam, Robin, & Vincent, 2009; Pulvers & Watson, 2017). Many factors have been shown to influence the prehospital time, including personal demographic factors, such as age, gender, education, income, and place of residence (Pulvers & Watson, 2017; Song et al., 2015; Zhou et al., 2016); clinical factors, such as history of stroke or cardiovascular disease, patient health characteristics, stroke symptomatology, stroke etiology, the vascular area involved in the stroke, and stroke severity (Miriam et al., 2009; Pulvers & Watson, 2017; Sobral et al., 2019; Sommer et al., 2017); cognitive and behavioral factors such as lack of attention to symptoms, stroke treatment awareness, patient and bystander behavior (Pulvers & Watson, 2017; Zhou et al., 2016); mode of transportation to the hospital, first visiting primary care facilities or a general practitioner, referral from another hospital, and others (Oostema, Konen, Chassee, Nasiri, & Reeves, 2015; Pulvers & Watson, 2017; Zhou et al., 2016). Presently, there is no available model for predicting the risk of prehospital delay after AIS. Such model would have the potential to significantly reduce onset‐to‐door (OTD) time and improve the outcome for patients with AIS. Considerable effort and expertise are required in the multidimensional analysis of prehospital delay, and more complex methods need to be developed to promote this complicated, preferably automated analysis (Wang, Wen, Lu, Yao, & Zhao, 2016).

Machine learning (ML) learns from observed data using a variety of artificial intelligence and statistical models to establish rational generalizations, discover patterns, classify unknown data, or predict new directions (Hosseinzadeh, Kayvanjoo, Ebrahimi, & Goliaei, 2013). ML methodologies, such as the Bayesian network (BN) and support vector machine (SVM), are being rapidly adopted in the medical field, because they enhance the practicability of classification and prediction. Prediction models are used for various diagnosis and prognosis tasks in the medical fields. This implementation may contribute to find ways to reduce drug costs, improve clinical researches, and promote better evaluation by physicians (Wang et al., 2016).

ML has been applied to predict the risk of death and functional outcomes after stroke (Park, Chang, & Nam, 2018). However, no published research used ML to predict prehospital delay after stroke. This study aimed to recognize the factors influencing the OTD time, compare the performance of logistic regression (LR)‐, BN‐, and SVM‐based models for prediction of prehospital delay after AIS, and develop a precise and effective model to predict the risk of prehospital delay in high‐risk populations that require intervention in order to shorten the time to medical treatment in these patients, thereby reducing the delay and enabling patients to receive timely and effective treatment.

## 2 METHODS

### 2.1 Study design and population

A cross‐sectional survey was conducted with a convenience sample of patients from a tertiary hospital. Patients diagnosed with AIS, aged 18 years or older, who were hospitalized between 1 November 2018 and 31 July 2019 and who had undergone at least one brain scan by computed tomography or magnetic resonance imaging were included in the study. Patients diagnosed with transient ischemic attack or cerebral hemorrhage, those with symptom onset at nursing homes or hospitals, patients with cognitive impairment or inability to answer question, and those who could not define the time of each interval were excluded from this study.

### 2.2 Data collection

Following the study protocol, the hospital provided training for researchers. We reviewed the medical records of patients diagnosed with AIS. For the patients who met the inclusion criteria, the data on demographics, health conditions, medical history, laboratory analyses, neuroimaging results, and therapies were extracted from the medical records. Stroke subtypes were recognized based on the Oxfordshire Community Stroke Project classification. Subsequently, the patients were interviewed by two well‐trained investigators in the hospital wards.

To determine whether there was prehospital delay, we asked the patients to state the first time he/she or someone else noticed the symptom, the time he/she went to the hospital, the time he/she arrived at the hospital, and the time he/she was started on treatment, and we computed the delay time. If the time we calculated based on the patients’ reports was unequal to that listed in the medical records, the former was selected, because the attending physicians may not be as cautious as the investigators in terms of inquiry and recording the times. The last normal time was regarded as the onset time for patients who were uncertain of the time of symptom onset or had a wake‐up stroke.

### 2.3 Measurements

The questionnaire was designed to include known sociodemographic, clinical, cognitive, and behavioral factors. The questions were revised after pilot test with 30 questionnaires.

In addition, the Stroke Premonitory Symptoms Alert Questionnaire developed by Zhang et al. (2015) was used to measure how the patients judged and decided whether to seek medical treatment when premonitory symptoms of stroke occurred. This questionnaire consists of nine items. Using a two‐level scoring method, 1 point is assigned for the correct answer, and 0 points for the wrong answer. The higher the score of the questionnaire, the more alert the patients are to the premonitory symptoms of stroke.

The Stroke Knowledge Questionnaire designed by Yan and Yang (2017) was used to evaluate the patients’ knowledge about stroke. The questionnaire includes 6 dimensions of stroke symptoms, first aid treatment, risk factors, safe medication, healthy behavior style, and rehabilitation knowledge, with a total of 40 items. Each item is scored by a two‐point method, that is, correct answers are given 1 point and incorrect or not known answers are given 0 points. The total score ranges from 0 to 40 points.

### 2.4 Definition of prehospital delay

We defined prehospital delay as an onset‐to‐door time of 3 or more hours. National guidelines and consensus highly recommend intravenous rt‐PA thrombolysis to be applied to patients with AIS within 4.5 hr and door‐to‐needle time within 60 min (Kobayashi et al., 2017; Powers et al., 2018). A study in China reported that the mean in‐hospital delay is more than 60 min (Wang et al., 2011). Thus, we considered that 3 hr of prehospital delay would cause the missed optimal thrombolytic therapy time for patients with AIS. In fact, most studies on prehospital delay have used 3 hr as a threshold (Nepal et al., 2019; Zhou et al., 2016).

### 2.5 Data analysis

The data extracted from the medical records and completed questionnaires were double entered into EpiData (version 3.1). SPSS Statistics 25.0 and SPSS Modeler 18.2.1 (IBM, Armonk, NY, USA) were used for statistical analysis. Patients were divided into timely admission group (OTD < 3 hr) and delayed admission group (OTD ≥ 3 hr). Quantitative variables were expressed as mean and standard deviation and comparative analysis was conducted using Student's *t* test. Qualitative variables were expressed as numbers and percentages, and comparative analysis was performed using the chi‐square test or Fisher's exact test.

### 2.6 Construction of the prediction models

In this study, the LR, BN, and SVM models were constructed using SPSS Modeler, and the default values in Modeler were applied for all unspecified parameter values. In addition, we used a 10‐fold cross‐validation to validate each prediction model, in which the dataset was divided into 10 equal‐sized parts, then trained with nine parts and tested with one part. We repeated the process until all data had been tested. Moreover, 10‐fold cross‐validation was repeated 10 times to avoid the randomness of cross‐validation and we used the mean as the final result (Cui, Wang, Wang, Yu, & Jin, 2018).

#### 2.6.1 LR

Since the risk prediction model in this study targets with two discrete categories (timely/delayed), the option “Binomial” was selected in the logistic regression “Procedure” of the SPSS Modeler.

#### 2.6.2 BN

BN was designed for prediction and classification based on the Bayes theorem (Friedman, Dan, & Goldszmidt, 1997). BN can intuitively encapsulate the causal relationship between factors stored in the medical data; therefore, it is widely used for medical decision support (Park et al., 2018). In addition, due to the characteristics of conditional probabilities and logical inherence in decision support, BNs can provide interpretable classifiers (Letham, Rudin, McCormick, & Madigan, 2015). Moreover, any given node could be queried in BNs, which are more clinically practical than classifiers built based on specific outcome variables (Park et al., 2018).

There are two possible selections of structure for constructing BNs: Markov Blanket and Tree Augmented Naive Bayes model (TAN). In order to find the most appropriate structure for predicting prehospital delay, we considered and compared the performance of BN models with different structures. Additionally, “Bayes adjustment for small cell counts” was chosen for parameter learning method.

#### 2.6.3 SVM

SVM is an ML algorithm with a good regularization attribute that is based on the structural risk minimization principle of statistical learning (Xiang et al., 2019). The SVM optimization process maximizes prediction accuracy and reduces over‐fitting of training data (Lotfnezhad, Ahmadi, Roudbari, & Sadoughi, 2015).

To improve the performance of the SVM model, we set the parameters as follows: The kernel function was the radial basis function with parameters C = 5 and γ = 0.161 (Xiang et al., 2019). The weights between generalization error and empirical error were represented by parameter C, and the shape of the separated hyperplane was controlled by parameter γ (Wang et al., 2016).

### 2.7 Prediction process

**FIGURE 1:**
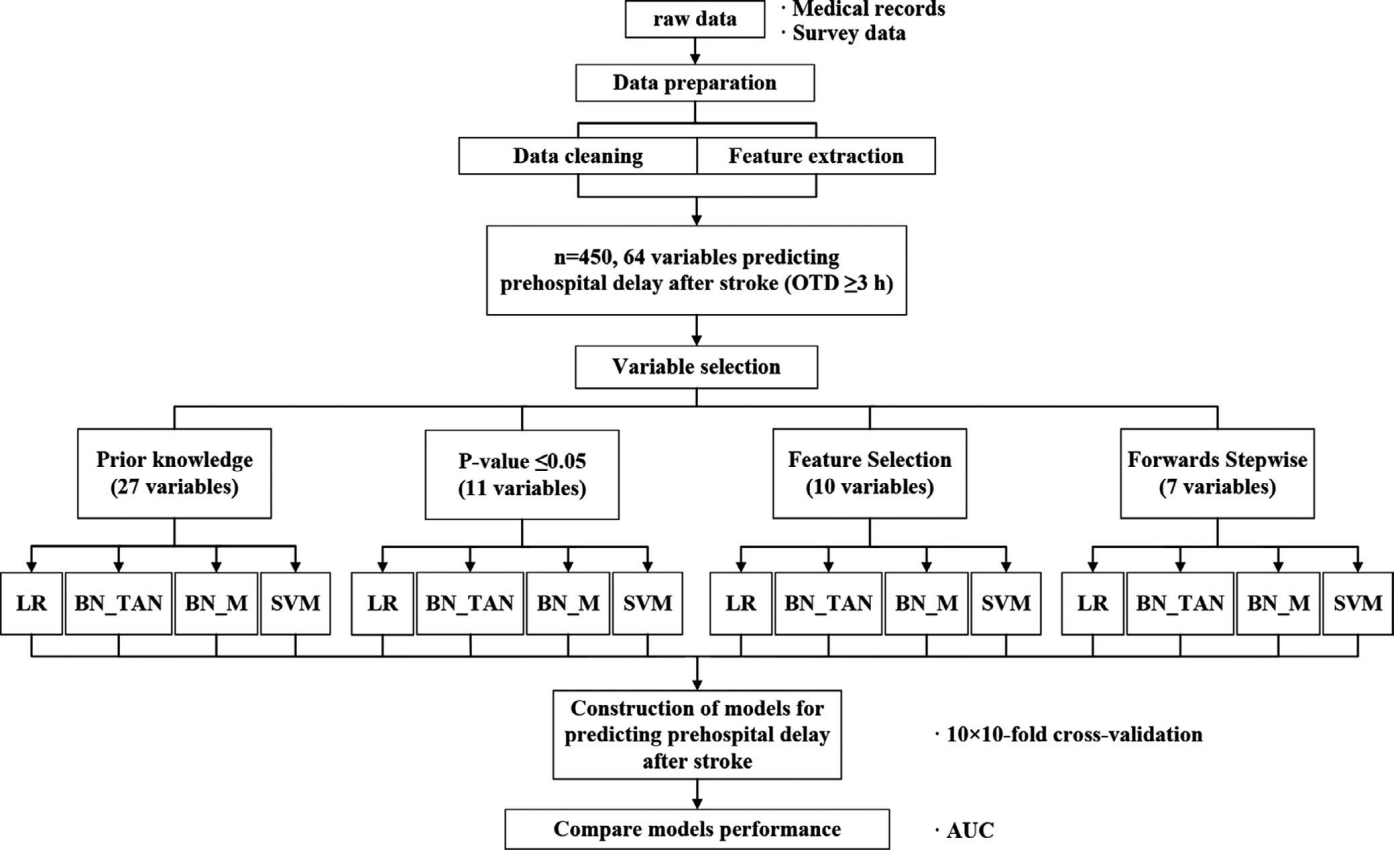
Process of constructing models for predicting prehospital delay after stroke. AUC, the area under the curve of the receiver operating characteristic; BN_M, Bayesian networks built by Markov Blanket structure; BN_TAN, Bayesian networks built by Tree Augmented Naive Bayes model structure; LR, logistic regression; OTD, onset‐to‐door time; SVM, support vector machine

The whole process of establishing a model to predict prehospital delay for patients with AIS is shown in Figure 1. We extracted 64 variables from the dataset and implemented a data preparation process to filter the records that met the exclusion criteria or lacked outcome variables. Considering the practicability of the model (predicting the possibility of prehospital delay for high‐risk groups), variables that are not suitable for prediction were not included in the model construction.

Considering that the performance of the model is largely dependent on choosing the appropriate variables, four different variable selection methods were tested for model construction in this study. We first selected 27 variables based on expert opinions and previous studies (called “Prior knowledge”), a classical method that is still widely used (Van Os et al., 2018). In addition, three more variable selection methods were considered: (a) variables with a *p*‐value ≤ .05 were included in the model; (b) feature selection, a method of identifying the most important variables for prehospital delay. Importance was ranked based on the likelihood‐ratio chi‐square and variables with importance greater than 0.95 were selected. We used the “Feature Selection Node” in SPSS Modeler to implement this operation; and (c) selection based on logistic regression forwards stepwise.

Next, we used LR, BN, and SVM to develop models for discriminating between the timely admission and delayed admission groups. Each prediction model was validated by 10 × 10‐fold cross‐validation. The discriminations of all LR, BN, and SVM models were compared. We adopted the area under the receiver operating characteristics curve (AUC) to assess the models’ performance (Hosmer & Lemeshow, 2013). Because of the 10 × 10‐fold cross‐validation, we gained 100 results for each model. We calculated the mean AUC for all models. In addition, we compared the performance of the optimal LR model with that of the optimal ML model by calculating the difference of the mean AUCs, including the relevant 95% confidence interval (CI).

### 2.8 Ethical statement

This study was approved by the Research Ethics Committee of Harbin Medical University, Harbin, China (Ref. No. KY2017‐063). The study purpose statement was read by all study subjects and each provided written informed consent. The patients’ identity information was kept confidential.

## 3 RESULTS

### 3.1 Statistical characteristics

Our study included 450 patients with AIS. Overall, only 12.7% of them presented to the hospital within 3 hr from onset. Table 1 shows the delay rates according to the patients’ characteristics and Table 2 shows the scores of the Stroke Premonitory Symptoms Alert Questionnaire and the Stroke Knowledge Questionnaire in the two groups. Notably, lower rates of delay were found in patients who lived in urban areas, who had higher incomes, those with commercial medical insurance, who underwent physical examination more than once a year, patients with a previous stroke, those whose onset location was at public or in the car, patients who had sudden symptoms, those with the following symptoms: speaking or understanding of speech difficulties, unilateral facial numbness or weakness, left arm weakness or numbness, and unconsciousness or fainting, patients who were aware of stroke and regarded the symptoms as serious, those who knew the time window for intravenous thrombolysis for stroke, patients who called emergency medical services instead of doing nothing, those who were accompanied by someone at the time of stroke onset, those in whom bystanders identified stroke and suggested that the patients go to hospital, patients with a distance between the place of onset and the investigating hospital smaller than 5 km, those who had used an ambulance before and this time, patients who had anterior circulation strokes, and patients who were more alert to the premonitory symptoms of stroke.

**TABLE 1:** Prehospital delay by patients characteristics (*n* = 450)

Factors	Total (%)	<3 hr (%)	≥3 hr (%)	*χ* ^2^	*p*	Variable name
Overall rate (*N*, %)	450, 100%	57, 12.7%	393, 87.3%			
*Sociodemographic and socioeconomic factors*						
Gender				1.097	.295	g1
Male	296	41 (13.9)	255 (86.1)			1
Female	154	16 (10.4)	138 (89.6)			2
Age, year				0.004	.947	g2
<65	322	41 (12.7)	281 (87.3)			1
≥65	128	16 (12.5)	112 (87.5)			2
Ethnicity				0.061	.805	g3a
Han nationality	418	52 (12.4)	366 (87.6)			1
Non‐Han nationality	32	5 (15.6)	27 (84.4)			2
Place of residence				15.775	**.000**	g4a
Rural	152	6 (3.9)	146 (96.1)			1
Urban	298	51 (17.1)	247 (82.9)			2
Occupation^a^				2.900	.089	g5a
Manual work	229	23 (10.0)	206 (90.0)			1
Nonmanual work	221	34 (15.4)	187 (84.6)			2
Number of children				1.202	.273	g7a
≤1	220	24 (10.9)	196 (89.1)			1
>1	230	33 (14.3)	197 (85.7)			2
Living				0.001	.975	g8a
Not alone	414	53 (12.8)	361 (87.2)			1
Alone	36	4 (11.1)	32 (88.9)			2
Education				0.010	.921	g9a
≤6 years^b^	116	15 (12.9)	101 (87.1)			1
>6 years	334	42 (12.6)	292 (87.4)			2
The monthly household income per capita (yuan)				12.636	**.005**	g10
<1,000	51	4 (7.8)	47 (92.2)			1
1,000–2,999	226	22 (9.7)	204 (90.3)			2
3,000–5,000	106	14 (13.2)	92 (86.8)			3
>5,000	67	17 (25.4)	50 (74.6)			4
Commercial medical insurance				4.439	**.035**	g11a
Yes	56	12 (21.4)	44 (78.6)			1
No	394	45 (11.4)	349 (88.6)			0
Medical insurance				0.024	.876	g11b
Yes	408	52 (12.7)	356 (87.3)			1
No	42	5 (11.9)	37 (88.1)			0
Family history of stroke				4.238	.120	b12
Yes	104	19 (18.3)	85 (81.7)			1
No	248	29 (11.7)	219 (88.3)			2
Unknown	98	9 (9.2)	89 (90.8)			3
Physical examination status				8.061	**.005**	b13a
≥1/year	113	23 (20.4)	90 (79.6)			1
<1/year	337	34 (10.1)	303 (89.9)			2
*Medical history*						
History of stroke				4.370	**.037**	b14a
Yes	115	21 (18.3)	94 (81.7)			1
No	335	36 (10.7)	299 (89.3)			0
History of hypertension				2.100	.147	b14c
Yes	244	36 (14.8)	208 (85.2)			1
No	206	21 (10.2)	185 (89.8)			0
History of diabetes				0.278	.598	b14d
Yes	99	11 (11.1)	88 (88.9)			1
No	351	46 (13.1)	305 (86.9)			0
History of hyperlipidemia				0.731	.393	b14e
Yes	64	6 (9.4)	58 (90.6)			1
No	386	51 (13.2)	335 (86.8)			0
History of coronary artery disease^c^				0.351	.554	b14f
Yes	82	12 (14.6)	70 (85.4)			1
No	368	45 (12.2)	323 (87.8)			0
History of cardiac arrhythmia				0.319	.572	b14h2
Yes	21	4 (19.0)	17 (81.0)			1
No	429	53 (12.4)	376 (87.6)			0
History of TIA				0.505	.477	b14i
Yes	20	1 (5.0)	19 (95.0)			1
No	430	56 (13.0)	374 (87.0)			0
Drinking				0.271	.603	b15a
Yes	180	21 (11.7)	159 (88.3)			1
No	270	36 (13.3)	234 (86.7)			0
Smoking				0.847	.357	b15b
Yes	227	32 (14.1)	195 (85.9)			1
No	223	25 (11.2)	198 (88.8)			0
*Onset circumstances*						
Onset location				24.804	**.000**	f16c
At home	306	36 (11.8)	270 (88.2)			1
At workplace	70	4 (5.7)	66 (94.3)			2
At public	54	9 (16.7)	45 (83.3)			3
On the car	10	6 (60.0)	4 (40.0)			4
Other	10	2 (20.0)	8 (80.0)			5
Wake‐up stroke				14.543	**.000**	f17
Yes	82	0 (0.0)	82 (100.0)			1
No	368	57 (15.5)	311 (84.5)			2
Symptoms found on Holidays				1.400	.237	day1
Yes	143	22 (15.4)	121 (84.6)			1
No	307	35 (11.4)	272 (88.6)			2
Onset time				2.674	.102	f18b
Daytime (06:01–22:00)	358	50 (14.0)	308 (86.0)			1
Night (22:01–06:00)	92	7 (7.6)	85 (92.4)			2
Bystander				0.000	.994	f19
Yes	363	46 (12.7)	317 (87.3)			1
No	87	11 (12.6)	76 (87.4)			2
Who noticed the symptoms first				3.246	.072	f20a
Patient	363	51 (14.0)	312 (86.0)			1
Someone else	87	6 (6.9)	81 (93.1)			2
Symptom onset				12.228	**.000**	f21
Sudden	313	51 (16.3)	262 (83.7)			1
Gradual	137	6 (4.4)	131 (95.6)			2
Language impairment				5.299	**.021**	f22a
Yes	228	37 (16.2)	191 (83.8)			1
No	222	20 (9.0)	202 (91.0)			0
Unilateral facial numbness or weakness				5.790	**.016**	f22b
Yes	122	23 (18.9)	99 (81.1)			1
No	328	34 (10.4)	294 (89.6)			0
Bilateral facial numbness or weakness				5.263	**.022**	f22c
Yes	70	3 (4.3)	67 (95.7)			1
No	380	54 (14.2)	326 (85.8)			0
Left arm weakness or numbness				4.304	**.038**	f22d
Yes	158	27 (17.1)	131 (82.9)			1
No	292	30 (10.3)	262 (89.7)			0
Right arm weakness or numbness				0.050	.822	f22e
Yes	140	17 (12.1)	123 (87.9)			1
No	310	40 (12.9)	270 (87.1)			0
Left leg weakness or numbness				3.681	.055	f22f
Yes	140	24 (17.1)	116 (82.9)			1
No	310	33 (10.6)	277 (89.4)			0
Right leg weakness or numbness				0.539	.463	f22g
Yes	139	20 (14.4)	119 (85.6)			1
No	311	37 (11.9)	274 (88.1)			0
Blurred vision in one or both eyes				0.278	.598	f22hi
Yes	61	9 (14.8)	52 (85.2)			1
No	389	48 (12.3)	341 (87.7)			0
Headache				0.000	1.000	f22l
Yes	21	3 (14.3)	18 (85.7)			1
No	429	54 (12.6)	375 (87.4)			0
Unconsciousness or fainting				10.240	**.016**	f22m
Yes	5	3 (60.0)	2 (40.0)			1
No	445	54 (12.1)	391 (87.9)			0
Dizziness, loss of balance, difficulty in walking, or coordination				4.369	**.037**	f22kno
Yes	224	21 (9.4)	203 (90.6)			1
No	226	36 (15.9)	190 (84.1)			0
Symptom change before admission				44.149	**.000**	f23
No change	129	27 (20.9)	102 (79.1)			1
Exacerbated	178	12 (6.7)	166 (93.3)			2
Lightened	27	12 (44.4)	15 (55.6)			3
Fluctuated	116	6 (5.2)	110 (94.8)			4
Considered any kind of the symptoms as severe				36.632	**.000**	f24
Yes	196	46 (23.5)	150 (76.5)			1
No	254	11 (4.3)	243 (95.7)			2
Recognized the problem as stroke				25.652	**.000**	f27
Yes	151	36 (23.8)	115 (76.2)			1
No	299	21 (7.0)	278 (93.0)			2
People around recognized the problem as stroke				26.530	**.000**	f28
Yes	169	39 (23.1)	130 (76.9)			1
No	146	9 (6.2)	137 (93.8)			2
Unknown	135	9 (6.7)	126 (93.3)			3
Patient's response when symptoms first appeared				63.820	**.000**	x31
Contacted relative/acquaintance	123	27 (22.0)	96 (78.0)			1
Call a doctor	2	1 (50.0)	1 (50.0)			2
Went to hospital directly	71	17 (23.9)	54 (76.1)			3
Take medicine by themselves	67	2 (3.0)	65 (97.0)			4
Called emergency number (120)	9	6 (66.7)	3 (33.3)			5
Did nothing	178	4 (2.2)	174 (97.8)			6
Reactions of people around				21.236	**.000**	x32a
Suggested to go to hospital	328	56 (17.1)	272 (82.9)			1
Other	122	1 (0.8)	121 (99.2)			2
*Previous understanding of stroke*						
Had similar symptoms before				0.011	.917	f25
Yes	121	15 (12.4)	106 (87.6)			1
No	329	42 (12.8)	287 (87.2)			2
Received stroke education				1.341	.247	f26a
Yes	44	8 (18.2)	36 (81.8)			1
No	406	49 (12.1)	357 (87.9)			2
Knowing someone who had stroke				3.787	.052	f26b
Yes	238	37 (15.5)	201 (84.5)			1
No	212	20 (9.4)	192 (90.6)			2
Previous stroke experience				0.527	.468	f26c
Yes	109	16 (14.7)	93 (85.3)			1
No	341	41 (12.0)	300 (88.0)			2
Don't know the stroke				0.570	.450	f26d
Yes	146	16 (11.0)	130 (89.0)			1
No	304	41 (13.5)	263 (86.5)			2
Knowledge about time window of intravenous thrombolysis for stroke				43.640	**.000**	f29a
≤3 hr	32	13 (40.6)	19 (59.4)			1
≤4.5 hr	17	7 (41.2)	10 (58.8)			2
≤6 hr	60	14 (23.3)	46 (76.7)			3
Unknown	341	23 (6.7)	318 (93.3)			4
*Transportation*						
Referred from other hospital				11.351	**.001**	change
Yes	133	6 (4.5)	127 (95.5)			1
No	317	51 (16.1)	266 (83.9)			2
Distance between the place of onset and the investigating hospital				35.363	**.000**	x35a
≤5 km	20	8 (40.0)	12 (60.0)			1
>5 km and ≤10 km	41	9 (22.0)	32 (78.0)			2
>10 km and ≤20 km	67	17 (25.4)	50 (74.6)			3
>20 km	322	23 (7.1)	299 (92.9)			4
Transportation means to first hospital				19.278	**.000**	x36b
Car	301	33 (11.0)	268 (89.0)			1
Ambulance	24	10 (41.7)	14 (58.3)			2
Other^d^	125	14 (11.2)	111 (88.8)			3
Have used an ambulance before				4.420	**.036**	x38
Yes	30	8 (26.7)	22 (73.3)			1
No	420	49 (11.7)	371 (88.3)			2
Have used an ambulance this time				23.019	**.000**	x39
Yes	31	13 (41.9)	18 (58.1)			1
No	419	44 (10.5)	375 (89.5)			2
Emergency personnel contact the hospital in advance.				1.352	.628	x41
Yes	5	2 (40.0)	3 (60.0)			1
No	14	4 (28.6)	10 (71.4)			2
Unknown	12	6 (50.0)	6 (50.0)			3
Traffic jam during transit				0.855	.652	x42
Yes	145	21 (14.5)	124 (85.5)			1
No	282	34 (12.1)	248 (87.9)			2
Unknown	23	2 (8.7)	21 (91.3)			3
*Clinical factors*						
OCSP classification				0.499	.480	type1
Lacunar	355	47 (13.2)	308 (86.8)			1
Nonlacunar	95	10 (10.5)	85 (89.5)			2
Vascular area involved in the stroke				19.120	**.000**	type2
Anterior circulation strokes	185	38 (20.5)	147 (79.5)			1
Posterior circulation strokes	236	19 (8.1)	217 (91.9)			2
Unknown	29	0 (0.0)	29 (100.0)			3
NIHSS score				2.369	.124	score1b
<4	286	31 (10.8)	255 (89.2)			1
≥4	164	26 (15.9)	138 (84.1)			2

The bold value means *p*‐value ≤.05, indicating that the difference is statistically significant.

Abbreviations: NIHSS, National Institutes of Health Stroke Scale; OCSP, Oxfordshire Community Stroke Project.

^a^ Manual includes those engaged in construction, farming/forestry/fishing and related, installation and related, manufacture and production, transportation and driver occupations; Nonmanual includes management, service, professional, commercial, and administration.

^b^ Defined as illiterate or having only finished primary education.

^c^ Coronary artery disease includes prior myocardial infarction or angina.

^d^ Defined as bicycle/tricycle or other vehicles.

**TABLE 2:** Compare the scores of Stroke Premonitory Symptoms Alert Questionnaire and Stroke Knowledge Questionnaire in two groups

Factors	<3 hr (*n* = 57)	≥3 hr (*n* = 393)	*p*	Variable name
Stroke aura symptom alertness	7.18 ± 2.001	6.58 ± 2.352	**.043**	cz
Stroke knowledge	31.93 ± 5.976	30.94 ± 5.238	.191	score2

The bold value means *p*‐value ≤.05, indicating that the difference is statistically significant.

### 3.2 Variable selection results

According to the results of the variable selection (Table 3), the logistic regression forwards stepwise filter method had a better model performance and the number of variables selected by this method was the least. Therefore, the seven variables selected by forwards stepwise filter method were used as the optimal variable subset for model construction. The selected variables were ranked according to the importance: patient's knowledge about time window of intravenous thrombolysis for stroke (0.30), patient's response when symptoms first appeared (0.26), referred from other hospital (0.15), place of residence (0.13), knowing someone who had stroke (0.10), age (0.06), and number of children (3.30E‐8).

**TABLE 3:** Results of variable selection

Filter method	Number of variables	AUC	*SE*	95% CI	
				Lower	Upper
Logistic regression					
Prior knowledge	27	0.788	0.011	0.766	0.809
*p* value ≤ .05	11	0.814	0.010	0.795	0.834
Feature selection	10	0.826	0.010	0.807	0.845
Forwards stepwise	7	0.846	0.009	0.828	0.864
Bayesian networks built by TAN structure					
Prior knowledge	27	0.773	0.009	0.754	0.792
*p* value ≤ .05	11	0.773	0.011	0.752	0.794
Feature selection	10	0.808	0.009	0.790	0.827
Forwards stepwise	7	0.832	0.010	0.813	0.851
Bayesian networks built by Markov Blanket structure					
Prior knowledge	27	0.782	0.010	0.762	0.803
*p* value ≤ .05	11	0.777	0.011	0.755	0.798
Feature selection	10	0.782	0.011	0.761	0.804
Forwards stepwise	7	0.800	0.010	0.780	0.819
Support vector machine					
Prior knowledge	27	0.777	0.009	0.759	0.795
*p* value ≤ .05	11	0.769	0.011	0.747	0.790
Feature selection	10	0.773	0.010	0.752	0.794
Forwards stepwise	7	0.801	0.009	0.783	0.819

Note

Model performance is assessed by calculating mean area under the curve (AUC) of the receiver operating characteristic across all cross‐validation folds.

Abbreviations: CI, confidence interval; *SE*, standard error; TAN, Tree Augmented Naive Bayes model.

### 3.3 Comparison among prediction models

The predictive performance of the four models is shown in Table 3. All models composed of the seven variables selected by forwards stepwise provide excellent discrimination, with mean AUCs ranging from 0.800 to 0.846. In addition, our results indicated that BN model built by TAN structure (BN_TAN) (mean AUC: 0.832) had a better diagnostic capability than BN model built by Markov Blanket structure (BN_M) (mean AUC: 0.800). The optimal ML model (BN_TAN, mean AUC: 0.832) and the optimal LR model (mean AUC: 0.846) had a similar discriminative power in predicting prehospital delay (difference of mean AUCs: 0.014; 95% CI: 0.013–0.015).

## 4 DISCUSSION

It has been proven that intravenous thrombolysis with rt‐PA is highly effective in reducing irreversible brain damage, preventing death, and improving the long‐term prognosis (Wardlaw et al., 2014). Controlled multicenter studies showed that the best time for administration of alteplase is no more than 3 hr, and it is also useful for patients with AIS treated within 4.5 hr (Emberson et al., 2014; Lees et al., 2016). Time is of uttermost importance and this may be the reason why patient's knowledge about the time window of intravenous thrombolysis for stroke was the strongest predictor of prehospital delay. In fact, previous studies reported that the knowledge about thrombolysis was independently associated with a lower rate of prehospital delay (Pulvers & Watson, 2017; Yanagida, Fujimoto, Inoue, & Suzuki, 2014). In addition, our results revealed that in 39.6% of the patients, the initial reaction was doing nothing and waiting for the symptoms to disappear, and the OTD time of these patients was longer than that of patients whose first reaction was to make emergency calls, go to hospital directly, or seek help from other people. Faiz, Sundseth, Thommessen, and Rønning (2014) and Zhou et al. (2016) also found that patients who hold a wait‐and‐see attitude and waited for their symptoms to relieve were prone to arrive late. Prior studies have indicated that referral from another hospital was one of the top three factors related to prehospital delay (Pulvers & Watson, 2017). In this study, we also discovered that referral from another hospital would contribute to a long OTD time. Moreover, Yang et al. (2014) and Zhou et al. (2016) indicated that the place of residence was the major factor influencing prehospital delay, which was also found in our research. One study found that patients with relatives or friends suffered a stroke may be more concerned about stroke symptoms and better understand the importance of admission immediately after onset (Zhou et al., 2016). Consistent with the findings of previous studies (Jin et al., 2012; Song et al., 2015), we found that advanced age was related to shorter prehospital delays. This may be due to younger patients not having a sense of urgency, while older patients are more likely to interpret symptoms as stroke and treat them as emergencies (Pulvers & Watson, 2017). It has also been reported that the lack of company when stroke symptoms occur for the first time may increase the prehospital delay (Jin et al., 2012; Zhou et al., 2016). In addition, we found that patients with less than one child are at higher risk of being alone at the time of stroke onset. Therefore, the patient's response when symptoms first appeared, referral from other hospital, place of residence, knowing someone who had stroke, age, and number of children were selected as predictors of prehospital delay after stroke. Furthermore, the results may indicate that excepting demographic variables in the electronic database, healthcare facilities should also consider collecting social and behavioral variables that are easily available in daily work, which could further improve the practicality and generalizability of prediction models.

In this study, we constructed models for prehospital delay prediction after stroke based on routine available medical records and survey data, and in order to enable the model to be used in high‐risk populations, the variables we used are all available before stroke onset. We found that the optimal ML model and the optimal LR model performed similarly in predicting prehospital delay after stroke. For prediction of OTD ≥ 3 hr using variables available in high‐risk people, all models performed good discrimination. This may reveal that prehospital delay of stroke depends on the presence of features in the variables selected by logistic regression forwards stepwise, such as whether patients know of the time window for intravenous thrombolysis after stroke, their response when symptoms first appear and whether they are referred from another hospital.

It was anticipated at first that the ML (BN and SVM) would outperform the LR models because they can evaluate a large number of variables at the same time and deal with nonlinear relationships and their interactions more efficiently. However, there was no significant difference in the discrimination of prehospital delay between the best ML model and the best LR model, although there were a large number of variables (64 in total) in the database available for analysis. This may be due to the use of multiple variable filter methods, and ultimately, only seven variables were involved in the final model construction. Moreover, this may indicate that nonlinear relationships and interactions are of limited importance in our dataset. Nevertheless, we found that ML served as an effective alternative to conventional LR in identifying the key variables. And we speculate that the strengths of applying ML algorithms to the prediction of prehospital delay may be more fully verified in a larger population since ML, when compared to traditional statistical methods, has advantages in handling large scale and high dimensional datasets.

The strengths of this study include the standardized collection of patient data and variable selection methods. In many studies, ML algorithms were compared with LR only using variables selected by prior experience, and the performance of ML was better than that of LR (Decruyenaere et al., 2015; Kop et al., 2016). Since the selection of variables is strictly in accordance with expert opinion and literature, the main disadvantage of variable selection by prior knowledge is that the prediction modes in the data might be lost (Van Os et al., 2018). In our research, compared to using other variable selection methods, the performance of LR using variables based on prior knowledge was poor. In addition, ML algorithms are very flexible and tend toward overfitting, leading to optimistic predictive performance; to compensate for this, we used 10 × 10‐fold cross‐validation, which is regarded as an effective method (Krstajic, Buturovic, Leahy, & Thomas, 2014). Moreover, the cross‐validation showed that each model had 100 performances and we computed the mean performance to compare the advantages and disadvantages of the models, which makes our results more reliable.

The reasons behind a prediction need to be understandable by physicians and patients; thus, for ML models in the medical field that interpretability is a core requirement (Park et al., 2018). Due to the high incidence, high mortality, high disability, and low thrombolysis rates of stroke, studying prehospital delay in patients with stroke are critical for both policy development and clinical care. Therefore, the prediction models of prehospital delay need to meet the requirements of high specificity and interpretable results. The advantage of LR is that it is transparent for each variable coefficient, because the odds ratio could be derived from these coefficients (Van Os et al., 2018). SVM and LR are similar in calculating a set of variable coefficients according to the transformation of the feature space (Wang et al., 2016). They differ mainly in that LR tries to define the probability of the modeling results (through the odds), while SVM intends to seek out the optimum dividing hyperplane directly, regardless of the real probability of category membership (Wang et al., 2016). The connections between variables in medical data can be intuitively established by BN, and in medical decisions, it could provide interpretable determinations (Park et al., 2018). Therefore, the BN is quite suitable for indicating the causality and uncertainty in predicting prehospital delay for patients after stroke. Furthermore, the construction of BN can use incomplete and partially correct statistics for medical diagnosis or prediction, since it is based on the conditional probabilities between variables to determine causes and effects (Park et al., 2018).

To the best of our knowledge, this study is the first attempt to apply ML algorithms as a supplement and alternative to the traditional statistical methods to recognize the factors influencing the OTD time and construct prediction models for prehospital delay in high‐risk populations for stroke. In this research, all the LR, BN, and SVM models were built only by SPSS and SPSS Modeler. It is well known that due to its user‐friendliness, SPSS is extensively applied in the medical field. Compared with other software, it is easier for healthcare personnel to use models in SPSS (Wang et al., 2016). ML models provide new possibilities for discovering health‐related factors that remain hidden in traditional analytical approaches. We applied ML technologies as a complement to LR to develop predictive models of prehospital delay for populations with a high risk of stroke. Our research can be used as healthcare data to develop new clinical assessments and interventions for these populations. That is, it will be possible to develop prehospital delay measurement tools specifically targeted at high‐risk groups for stroke, which can help prioritize interventions for risk groups. In addition, according to the identified influencing factors, this study could also help healthcare personnel to provide guidance to patients to reduce the OTD time, thereby helping patients to receive timely and effective treatment.

## 5 LIMITATIONS AND FUTURE DIRECTION

There are several limitations in the present study; prospective studies in predictive modeling need to be improved.

First, convenience samples may be biased because individuals who choose to participate in the study may not fully represent the population from which the sample has been drawn. Nevertheless, this choice was justified as it provided a representative sample of AIS. A low response rate is a limitation. Declining response rates have been recognized among patients. Poor rates cause nonresponse bias, which may seriously affect the validity of the study in terms of generalizability and applicability of the findings. However, this study well exceeded the required number of responses estimated by power analysis providing representative samples of AIS patients.

Second, we only examined the effects of individual variables but we did not study the relation between variables and the nature of direct or indirect influencing factors. In the future, it is necessary to study how variables affect predictability through detailed univariate analysis and identification of the meaning.

Third, in our research, we used the same data as the training data and test data used for cross‐validation. In the future, in case of a larger sample size, we will ensure that the training data differ from the test data in advance to obtain more exact results.

How to implement these models in the clinical practice is an important question we need to solve in the future. In order to promote prehospital delay prevention, we can develop a user‐friendly, foolproof, web‐based clinical support system based on optimal ML algorithms to achieve “real‐time individualized feedback,” which can be accessed by means of mobile devices or personal computers. This universal design could facilitate and promote use in busy clinical settings including visits to out‐patient clinics, in‐patient consultation, or quick assessment by nonphysician users. For healthcare professionals, identification of patients who prone to prehospital delay after stroke has the potential to enhance disease control and management by allowing for tailored interventions which significantly improve the allocation of social‐related resources. From the standpoint of policy makers, the system provides a method with less expense to conduct a beneficial evaluation.

## 6 CONCLUSION

In this study, we identified the important factors that affect the early admission of patients with stroke and we evaluated the performance of LR, BN, and SVM models in predicting prehospital delay after stroke. We found that ML algorithms were not inferior to conventional LR in recognizing the key variables, thus creating a valuable diagnostic procedure for prehospital delay prediction in high‐risk groups for stroke. For these models to be used in daily routine, some work still needs to be done, nevertheless, this work opens new lines of research.

## CONFLICT OF INTEREST

The authors have no conflicts of interest to report.

## AUTHOR CONTRIBUTION

LY, QL, and QZ contributed conception and design of the study. QL organized the database and performed the statistical analysis. LY wrote the manuscript. LY, QL, QZ, XZ, and LW contributed to data interpretation and revising the manuscript.

### PEER REVIEW

The peer review history for this article is available at https://publons.com/publon/10.1002/brb3.1794.

## Data Availability

The data that support the findings of this study are available from the corresponding author upon reasonable request.
